# Highlights on Drug and Ion Release and Recharge Capacity of Antimicrobial Removable Prostheses

**DOI:** 10.1055/s-0042-1758788

**Published:** 2022-12-27

**Authors:** Reem Abualsaud, Mohammed M. Gad

**Affiliations:** 1 Department of Substitutive Dental Sciences, College of Dentistry, Imam Abdulrahman Bin Faisal University, Dammam, Saudi Arabia

**Keywords:** removable dental prostheses, PMMA, candidiasis, mineralization, recharge, dental caries, fluoride release, caries prevention, antimicrobial effect, denture stomatitis

## Abstract

This article aimed to review the ion and drug release, recharge abilities, and antimicrobial properties of drug/ion-releasing removable prostheses, and to assess their capability in preventing and inhibiting denture stomatitis as well preventing caries and reversing carious lesions. Data was collected from published scientific papers listed in PubMed database from January 1975 to December 2021. English full-text articles, involving clinical or *in vitro* studies, focusing on removable prostheses and are concerned with drug/ion release and rechargeability as a way to prevent or inhibit denture stomatitis or dental caries were included. The relevant articles reported that ion- or drug-modified polymethylmethacrylate acts as a reservoir for these ions and drugs and is capable of releasing significant amounts with sustained release effect. Recharging of modified resin resulted in greater sustainability of ion and drug release, thus improving the long-term effects of protection against demineralization and reducing the adhesion of *Streptococcus mutans* and *Candida albicans* . Modifications of removable prostheses with rechargeable ions and drugs enhance remineralization, hinder demineralization, and reduce microbial adhesion in difficult-to-access areas. Selection of denture base for clinical use will consider its ability to act as an ion/drug reservoir that is capable of release and recharge.

## Introduction

Removable complete prostheses (RCPs) and removable partial prostheses (RPPs) are used to rehabilitate the mouths of geriatric patients with complete or partial edentulism, respectively. 1 These prostheses although considered valid treatment modalities, they are not without drawbacks. The microbial adhesion onto removable prostheses surfaces has been established as the first step for microbial growth and colonization. 2 Microbial adhesion to polymethylmethacrylate (PMMA) denture base material is affected by the material's wettability, surface topography, and degree of roughness, surface coating with saliva, and microbial surface charge. 2 3 Out of these factors, surface roughness was linked with the early stages of biofilm formation. 3 To minimize the side effects of microbes-infiltrated PMMA dentures and treat oral candidiasis, various antifungal agents have been developed and painted onto the surface. However, extended use of these antimicrobial drugs can lead to the emergence of new microbial strains with drug resistance. 4

In patients with RPPs, there is an additional risk of caries of the remaining teeth and especially the exposed roots of abutments with gingival recission. 5 The formation of microbial-loaded plaque around teeth or oral appliances increases the chance of carious lesion formation. 6 7 The presence of a denture base around teeth or on top of oral mucosa impedes normal oral cleansing mechanism or proper hygiene procedures of covered areas, reduces salivary flow around abutments, and facilitates plaque formation and demineralization. 7 8 Caries is usually initiated by the adhesion of cariogenic bacteria to teeth surfaces or oral appliances. If bacterial adhesion to tooth structure was hindered, then a reduction in the incidence of carious lesions may be anticipated. Other methods to prevent caries development include the use of fluoride-containing mouth rinses, fluoridated toothpaste, fluoride gels or varnishes, and sustainable source of fluoride ions such as fluoride-releasing restorative materials or devices. 3 9 10 11 For patients using RPPs, developing a fluoride-rechargeable denture base resin will significantly contribute to the reduction of root caries in abutment teeth. 5 For RCPs, microbial adhesion to denture bases resulted in tissue inflammation and denture stomatitis (DS) that may contribute to systemic disease exacerbation. 2 *Candida albicans* easily thrives in the oral cavities of the elderly due to prolonged use of dentures, 12 and has been reported to be the primary contributory microbe in oral candidiasis and other related infections, which encourages the development of oral antifungal therapies/dentures. 13 14

## Demineralization, Remineralization, and the Effect of Ions

Inhibition of enamel and dentin demineralization depends on oral fluoride concentration. It was reported that artificial saliva containing 2 ppm fluoride, calcium, and phosphate at a pH of 4.5 inhibited the demineralization of enamel, while fluoride levels above 1 ppm resulted in 40% inhibition of dentin demineralization. 15 The natural presence of fluoride within the oral fluids results in local action. Therefore, low concentrations of fluoride must be continually maintained in the oral cavity. 16 Fluoride's main function involves controlling the demineralization and remineralization of tooth structures. However, it has also been linked to modulation of the physiological processes of microbial cells, which can indirectly avert the formation of carious lesions. 3 17 Fluoride can dissolve in water and buffer the acidic condition produced by cariogenic bacteria, or promote remineralization to prevent carious lesions. 18 19 Additionally, fluoride ions available in the saliva or brought to the mouth through fluoride-containing dentifrices, mouth rinses, or gels, can be absorbed into the glassy matrix of teeth. 20 This process is not constant and fluctuates over time. 7 The anticariogenic mechanisms of fluoride include the inhibition of demineralization, enhancement of remineralization, formation of less soluble fluorapatite compounds, and reduction of microbial metabolism. 21 The degree to which caries was prevented was linked to fluoride level. 8

## Denture Stomatitis

The nature of denture PMMA makes it a suitable environment for the colonization of various microorganisms such as *C. albicans* , *Streptococcus mutans* , and *Staphylococcus aureus* . These microbes have been linked to numerous oral infections including DS, as well as systemic infections and pneumonia. 22 The prevention of *Candida* adhesion and biofilm formation on denture surfaces is the main step to prevent DS. 23 Systemic antibiotics have been used to treat oral infections, but their excessive use may lead to the formation of drug-resistant strains. 24 On the contrary, local use of topical antibiotics did not demonstrate high effectiveness due to the inability to maintain contact with oral tissues in addition to the continuous washout by saliva. Therefore, there is an essential need for a technique that will sustain the amount of drug released, in addition to control the site of delivery. 22 25 Also, direct and localized delivery of the drug prevents systemic side effects or drug–drug interactions. 22 Mechanical scrubbing is one of the primary methods to reduce plaque formation and lower the number of microorganisms on the surfaces of oral appliances. However, yeasts were found to have quite strong adhesion to denture surfaces. Additionally, several microorganisms were resistant to chemical disinfection. 26 Other methods have been developed to minimize fungal colonization and further biofilm formation. These include coating the denture surface with nontoxic polysaccharides, and modifying the denture base material with polyethylene oxide or phosphate. 3

### Candida Adhesion and Invasion

*C. albicans* is considered one of the main microorganisms of denture plaque and its adhesion to PMMA is attributed to two mechanisms (ionic charge and glycoproteins). 12 The involved gene-encoded glycoproteins are important for *candida* cell adhesion, host tissue penetration, and *candida* morphological transformation. Similarly, the gene family that encodes secreted aspartyl proteinases, is imperative for multiple pathogenic processes including host protein digestion, host tissue invasion, and avoidance of the host immune system. Their expression increases during the hyphal stage which advocates a role in hyphal formation. This confirms the importance of glycoproteins- and proteinase-related genes in determining which candida form would exist. 13

### Fungal Form (Hyphal Form versus Yeast Form)

*C. albicans* has the ability to morph into different forms depending on the surrounding conditions. 13 In normal conditions, it assumes the yeast form. However, it morphs into hyphal form after contacting the tissues and under the influence of surrounding serum conditions, temperature, pH, and nutrients (carbon, nitrogen, and amino acids). 13 It has been reported that contact with a solid surface and higher surface roughness enhances the yeast-to-hyphae transformation as a result of the thigmotropism of *candida* cells. 27 28 The yeast-to-hyphae transition involves the formation of longer germ tubes 29 that invade the surface defects and irregularities of the material. 27 The hyphal form has a higher pathogenic potential than the yeast form. 23 29 Therefore, to minimize the pathogenicity of *C. albicans* , biofilm formation should be stopped through the prevention of yeast-to-hyphae transition. 23 30 31 32

## Bioactive Materials

Bioactive restorative materials are those that bond to teeth and show an interaction at the tooth/restoration interface. They should positively affect the oral tissues and be a source of ions (fluoride, calcium, and phosphate) important for the remineralization process. 23 33 Fluoride is a crucial element that prevents caries by seizing the demineralization and accelerating the remineralization processes. Additionally, fluoride has some antimicrobial properties and has been incorporated into restorative materials due to its low toxicity at therapeutic doses. 33 Previous reports confirmed that proximal teeth surfaces in contact with fluoride-releasing restorations showed a lower incidence of caries attack compared with surfaces contacting nonfluoride-releasing restorations. 11 34 Additionally, a restoration with sustained release of a certain level of fluoride ion might have caries inhibition abilities on adjacent natural teeth. 35 Restorative resins containing bioactive fillers have excellent ion release and recharge abilities. 5 Moreover, vehicles or carriers for ion/drug release and recharge were suggested to be incorporated within RCP and RPP denture base resins. 14 22 Also, the high ratio of surface area to volume of fluoride-modified nanofillers within a restorative material could be of benefit in increasing the ion release. 36

### Ions

Table 1 summarized the different ions release and recharge as investigated in the literature. Different bioactive materials such as surface prereacted glass ionomer (S-PRG), 3 5 10 13 18 23 30 fluoridated glass fillers, 11 Kavitan, Fritex, and pure sodium fluoride (NaF), 7 and fluoride-releasing filler G018–090 8 were investigated for their ion release and/or recharge abilities. S-PRG is an ion-releasing bioactive filler that is formed by an acid-base reaction of polyacrylic acid and fluoroaluminosilicate glass. It is capable of releasing many ions including fluoride, strontium, sodium, aluminum, boric acid, and silicic acid. 19 23 Therefore, it can induce remineralization and buffer the acidic products of cariogenic bacteria. 19 23 S-PRG particles showed a cyclic pattern of ion recharge and release upon need, which is beneficial to strengthen the tooth structure, inhibit tooth demineralization, buffer against intraoral acids, and inhibit dental plaque adhesion. 13 19 23 Due to the ability to release and recharge fluoride, it (S-PRG) has been introduced into dental filling materials. 18 The level of released ion has to balance between effectiveness and absence of toxicity. 7 Previous microbiological studies confirmed the inhibitory effects of fluoride and boric acid on *candida* adhesion, 33 37 suggesting the ability of S-PRG filler to reduce *candida* adhesion to PMMA surfaces after the release of active ions. Therefore, PRG filler serving as a fluoride reservoir was introduced into denture base resin. It could induce remineralization, and prevent microbial adhesion and further plaque formation. The modified PMMA inhibited dentine demineralization in a dose-dependent fashion. 10 19 23 30 Adding to that, Mukai et al 10 recommended the inclusion of the highest S-PRG content within PMMA due to its dose-dependent action against dentin demineralization. Nakornchai et al 8 investigated the effect of the solvent's pH during G018–090 silanization process on the amount of released fluoride from the modified resin. The results confirmed significant differences in fluoride release between different groups (different pH). 8 Another study reported that denture base resin modified with stannous fluoride had antibacterial effects against *Fusobacteria* . 38

**Table 1:** Collected data about ions release and recharge

Author/Year/Type	Resin and prostheses	Modifying agent/filler	Tested properties	Ion measuring method/device	Ion	Recharge/solution	Study duration	Results and outcome
[5] Kamijo et al 2009 *In Vitro*	HP DB HP URBAN, clear pink, Shofu Inc.	S-PRG filler (0, 5, 10, 20, 30%)	Flexural strength, flexural modulus, fluoride release	Fluoride electrode fluoride-ion meter	F ^-^	F ^-^	Phase I- 15 days Phase II- 49 days	- PMMA/S-PRG releases significant amounts of fluoride after recharging - Modified PMMA may assist in root caries prevention of abutment teeth
[10] Mukai et al 2009 *In Vitro*	HP DB URBAN, clear pink, Shofu Inc.	S-PRG filler (0, 5, 10, 20, 30%)	Inhibition of dentin demineralization	Ability to inhibit lesions and demineralization process	Mineral profile	No	7 days	- PMMA/S-PRG can prevent demineralization and prevent root caries of abutment teeth
[3] Al-Bakri et al 2014 *In Vitro*	HP DB Urban, clear pink, C2, Shofu Inc.	Silane-coated fluoridated glass fillers- ultrafine GM35429 (0, 1, 2.5, 5, 10%)	Surface roughness (Ra) Wettability Candida adhesion	NS	No	No	Immediate	- Fluoridated glass fillers had microbial antiadhesive property with no adverse effect of acrylic surface properties
[11]. Al-Bakri et al 2014 *In Vitro*	HP DB Urban, Shofu Inc.	Silane-coated fluoridated glass fillers- ultrafine GM35429 (0, 1, 2.5, 5, 10%)	Fluoride release Rechargeability Flexural properties	Fluoride selective electrode	F ^-^	F ^-^ immersed in 5 mL of 5,000 ppm neutral NaF (Neutral Fluor 5000 Plus, Colgate, USA) for 5 minutes	43 days	- PMMA/fluoridated glass fillers released fluoride ion initially and after recharge - Flexural strength decreased as glass filler content increased
[23]. Tsutsumi et al 2016 *In Vitro*	HP DB Urban, clear light pink, Shofu Inc.	S-PRG filler (0, 5, 10, 20%)	Surface roughness Candida adhesion	Na ^+^ , Sr ^+2^ , SiO _3_^-2^ , Al ^+3^ , BO _3_^-3^ , using atomic emission spectroscopy F ^-^ using electrode connected to a fluoride ion meter	F ^-^	No	24 hours after immersion	- Fillers increases the surface roughness of modified PMMA but reduced the candida adhesion
[8] Nakornchai et al 2016 *In Vitro*	HP DB Triplex hot, Ivoclar Vivadent	Acidic-adjusted and nonadjusted pH of silanized fluoride-releasing filler G018–090 (20%)	Fluoride release Flexural strength Flexural modulus	Fluoride ion selective electrode	F ^-^	No	2 months	- Nonsilanized fillers released greatest fluoride content initially but showed lower flexural strength - Acidic-adjusted pH of solvent increased fluoride release, while nonadjusted pH showed better flexural strength
[30] Takakusaki et al 2018 *In Vitro*	Tissue Conditioner II, Shofu	S-PRG filler (0, 5, 10, 20%)	Candida adhesion Consistency and penetration depth Surface roughness	Fluoride ion electrode connected to fluoride-ion meter Na ^+^ , Sr ^2+^ , SiO ^3+^ , Al ^+3^ , and BO _3_^-3^ measured using plasma atomic emission spectroscopy	F ^-^	No	Immediate and 7 days	- All ions (Na ^+^ , Sr ^+2^ , SiO ^+3^ , Al ^+3^ , BO _3_^-3^ , F ^–^ ) were released from S-PRG/PMMA - Ion release was concentration dependent - S-PRG reduced *C. albicans* adhesion and affected the tissue conditioner properties - 10 wt% was the recommended concentration of loading
[18] Kiatsirirote et al 2019 Double blind randomized clinical trial	DB PMMA, URBAN Resin, Shofu Inc.	S-PRG filler (0, 20%)	Fluoride release Rechargeability	Fluoride ion meter	F ^-^	F ^-^ Fluoride toothpaste for 14 days. Followed by immersion in 1 g of 5,000 ppm fluoride gel/water for 1.5 years	1, 14, 15 days, 3 months, and 1.5 years	- 20 wt% S-PRG/PMMA released fluoride initially and for short duration - Recharge with fluoride ion increased the long-term release
[7] Raszewski et al 2021 *In Vitro*	Acrylic resin Superacryl Plus, SpofaDental	Two bioactive glass fillers: (0, 5, 10%) Kavitan plus powder and (0, 10%) Fritex, and (0, 10%) NaF	Fluoride ion release Rechargeability Sorption, and solubility	Spectrophotometer	F ^-^	F ^-^ Brushed with 1,450 ppm fluoridated toothpaste and stored in water for 10 hour	7, 14, 28, and 35 days	- 10 wt% Fritex or 10 wt% Kavitan improved PMMA properties - Fluoride release lasted for over 4 weeks

Abbreviations: HP DB, heat-polymerized denture base; NaF, sodium fluoride; NS, not stated; PMMA, polymethylmethacrylate; S-PRG, surface pre-reacted glass.

### Vehicle and Drug Carrier

Table 2 summarized different drug loading, release, and recharge as investigated in the literature. Drug nanocarrier must be progressively degradable, transport the drug to the target site, and deliver it in a controlled manner over time. In general, these drug carriers are either biodegradable or not. Biodegradable nanocarriers are versatile and include collagen, sponges and inorganic calcium phosphate nanoparticles. 8 Other drug carriers include N-vinyl-2-pyrrolidinone (NVP) monomer, 39 40 mesoporous silica nanoparticles (MSNs), 14 41 and hydroxyapatite nanoparticles (HA-NPs). 22

**Table 2:** Collected data about drug release and recharge

Author/Year/Type	Resin and prostheses	Modifying filler/carrier	Tested properties	Drug measuring method/device	Drugs	Recharge/Drug	Study duration	Results and outcome
[45] Amin et al 2009 *In Vitro*	Chemically activated PMMA, Paladur, Kulzer	Directly incorporated into PMMA	Drug release Antifungal effect	High-performance liquid chromatography (HPLC) Candida inhibition zone	Fluconazole (5%), chlorhexidine (5%), and combination of fluconazole + chlorhexidine (5/5%)	None	28 days	- All modified groups showed long-term (28 days) drug release and antifungal effect - Combination drugs showed the highest drug release and antifungal effect followed by chlorhexidine then fluconazole
[39]. Sun et al 2013 *In Vitro*	HP DB Lucitone 199, Dentsply Intl	Plasma-initiated graft-polymerization of N-vinyl-2-pyrrolidinone (NVP) at 0, 5, 10, 15, 20%	Water sorption and solubility Hydrophobicity/ hydrophilicity Flexural strength and modulus Drug binding Drug releasing (MIC) of drugs absorbed	NS Contact angle analyzer Mechanical testing system Beckman Coulter DU520 UV/Vis spectrophotometer UV Absorption Formation of candida inhibitory zone	Miconazole and chlorhexidine digluconate Charged with immersion in 5 wt% miconazole ethanol solution, or 5 wt% chlorhexidine digluconate solution overnight	Quenching by immersion in 5% PNVP solution for 8 hours Recharged by immersion in miconazole or chlorhexidine digluconate solution over night	60 days of miconazole release followed by quenching and recharge overnight with CD 14 days of CD release followed by quenching and recharge with miconazole	- Surface grafting with PNVP increased drug binding capacity - The antifungal effect of released drugs lasted for weeks to months - It is possible to wash-out loaded drug, recharge with the same or different drug
[14]. Lee et al 2016 *In Vitro*	Chemically activated orthodontic PMMA, Orthocryl resin, Dentarum	MSN (0.5, 1, 2.5, 5%) Amphotericin B loading of the 0 and 2.5% MSN	Flexural strength and modulus Surface hardness, energy, and roughness long-term antimicrobial effect Drug loading and release capacity	Universal testing machine Culturing of candida Absorbance at 416 nm	Amphotericin B	Amphotericin B loaded for 6 hours	Aging for 28 days after drug loading	- MSN-modified PMMA had superior mechanical properties and microbial antiadhesive affects - Amphotericin B can be loaded into MSN particles incorporated with PMMA to produce long-term antimicrobial effects
[40] Malakhov et al 2016 *In Vitro*	HP DB Lucitone199, Dentsply Intl	Plasma-initiated graft polymerization of PNVP Loaded with miconazole	Miconazole release and bioactivity chlorhexidine digluconate release	High-performance liquid chromatography (HPLC)	Miconazole and CD	Quenching by immersion in 5% PNVP for 8 hours To recharge: incubation in 5 wt% miconazole or 5 wt% CD	1, 2, 5, 10, 24, and 30 days	- Miconazole release was sustained for up to 30 days with greater availability at higher saliva concentration - Saliva does not interfere with miconazole release or its antifungal effect after recharge - Recharge of miconazole PNVP-PMMA with CD was effective and showed CD release up to 18 days
[41] Jo et al 2017 *In Vitro*	Chemically activated orthodontic PMMA, Orthocryl resin, Dentarum	MSN (0.5, 1, 2.5, 5%) MSN loaded by immersion in 1 mg/mL AgSD solution in acidic acetone-ethanol mixture	Long-term microbial antiadhesive effects AgSD loading Surface roughness, hardness, and wettability Flexural strength and modulus Amount of AgSD recharge Cytotoxicity	Inductively coupled plasma atomic emission spectrometry Thermogravimetric analyzer and UV/vis spectrometry Profilometer, Vickers hardness tester, and contact angle measurement Universal testing machine UV/vis spectrometry Water-soluble tetrazolium salt assay	AgSD	Recharge after aging for 28 days by incubating in AgSD-ethanol suspension for 1 hour	1, 2, 4, 7, 14, and 28 days after recharging with AgSD	- Ag-MSN modified PMMA showed long-term antimicrobial effects even after reloading of the drug - AgSD-MSN/PMMA presented superior flexural strength, hardness and antimicrobial properties up to 28 days - Aged specimens still had the potential to exert microbial antiadhesive effects
[22] Elboraey et al 2021 *In Vitro*	Chemically activated PMMA, Acrostone, dental and medical suppliers	HA-NP (0, 10%), metronidazole (0, 10%), and combination of HA-NP and metronidazole (10/10%)	Metronidazole release rate Antimicrobial properties against three Gram-positive pathogens Cytotoxicity Microhardness	UV/vis spectrophotometer MTT assay Vicker's hardness tester	Metronidazole	None	1 month	- HA-NP can be used a drug carrier and incorporated into PMMA material - HP-NP allows sustained drug release for the management of oral infections

Abbreviations: AgSD, silver-sulfadiazine; CD, chlorhexidine digluconate; HA-NP, hydroxyapatite nanoparticles; HP DB, heat-polymerized denture base; MSN, mesoporous silica nanoparticles; NS, not stated; PMMA, polymethylmethacrylate; PNVP, poly(N-vinyl-2-pyrrolidinone; UV/vis, ultraviolet-visible.

#### Poly(N-Vinyl-2-Pyrrolidinone)

The loading (charging) capacity of a drug carrier determines its ability to maintain a continuous antimicrobial effect. 14 Sun et al 39 produced poly(NVP) (PNVP)-grafted denture materials that can be reloaded with different drugs to prolong the antifungal effects. In this process, plasma-initiated polymerization was used to graft the NVP onto PMMA material. 39 Malakhov et al 40 grafted PNVP onto PMMA (PMMA-gPNVP), then loaded it with miconazole, and evaluated the drug release in solutions with different concentrations of human saliva. The results showed a sustained miconazole release for 30 days that was proportionate with saliva concentration. The PMMA-gPNVP disks showed similar drug release and activity after recharging with the same or different drug. 40

#### Mesoporous Silica Nanoparticles

MSNs, a Food and Drug Administration-approved biocompatible filler in the nanoscale with high stability, good durability, low cost, high surface area, high total pore volume, and capability of loading and recharging with other biomolecules have been used as a potential additive and drug carrier. 14 41 42 If used as nanoadditives in PMMAs, they have the potential to deliver microbial antiadhesive drugs. 41 42 As reported, PMMA/MSN loaded with amphotericin B was found to be an effective antimicrobial drug to prohibit fungal growth or infections. 14 PMMA loaded with 5 wt% MSNs, achieves long-term hydrophilicity-induced microbial antiadhesive effects (14 days) in combination with amphotericin B drug delivery. 14 However, the rechargeability of the modified PMMA/MSN and the long-term antimicrobial effects have not been fully explored, although they would be very useful for clinical use. 41

#### Hydroxyapatite Nanoparticles

Based on the idea that hydroxyapatite is a biocompatible and biodegradable naturally existent material in bones and teeth, Elboraey et al 22 aimed to develop biodegradable HA-NP that are capable of acting as carriers for metronidazole (MZ) within denture base resin in an attempt to manage oral infections. The results were promising in which there was a sustained release of MZ for up to 1 month. 22

## Antimicrobial Denture Concept

An alternative to the current treatment strategy for DS is to include the antifungal agent within the dentures. 39 43 44 This technique (antimicrobial denture) involves dispersing the antifungal drugs within the denture material and allowing it to dissolute from the device later to produce its antimicrobial effect. The highest concentration of the released antifungal drug is found within the surroundings of denture surfaces, generally surpassing the minimum concentration for microbial inhibition of susceptible species for a prolonged period (days to weeks). 39 Among the incorporated drugs are chlorhexidine, tetracycline, fluconazole, and amphotericin B, in addition to nanoparticles of silver, platinum, and copper. 44 45 Other additives have also been incorporated into polymers such as quaternary ammonium, tertiary amine, benzimidazole, and others, but did not show long-term (> 7 days) antimicrobial effects. 14 41 The major mechanism of antimicrobial agents is through modification of the surface energy and modulation (increase) in substrate surface hydrophilicity, resulting in microbial antiadhesive effects. The surface-free energy, topography, and amount of released ions depend on the incorporated nanocarriers within the PMMA. 14 If a sustained release of ions/drugs could be achieved, the concept of antimicrobial denture is a success.

### Sustained Release

An optimal characteristic of an antimicrobial removable prosthesis is the long-term antiadhesive property against microbial species. 14 The long-term effect ensures sustained antifungal ion/drug release for 30 or more days and allows rechargeability and re-release of a variety of drugs to customize the treatment depending on patient needs. 40

#### Ion Release: Time and Duration

A possible method of creating a denture base with antifungal leachable ions such as fluoride is to add fluoridated glass fillers, 11 or S-PRG into PMMA. 5 10 The released fluoride ions are produced after the dissolution of the inorganic fluoride contained within the added fillers into surrounding fluids. 11 46 Glass–ionomer matrix shows significant fluoride release initially, owing to the easily penetrable complex fluoride contained within. 18 The literature has also confirmed this finding for other materials. The glass-ionomer-based fissure sealants showed higher fluoride release initially and after recharge compared with other types of fissure sealants. 47 Studies reported a drop in the amount of released fluoride after immersion in distilled water following initial high levels. 5 46 Acrylic resin coated with fluoride ions showed significantly reduced adhesion of *S. aureus* and *S. mutans* ; however, the fluoride release was inconsequential and undetectable after 24 hours. On the other hand, fluoride dissolution from fluoridated fillers persisted for a longer duration reaching one-tenth the initial value after 7 days of immersion. 5 These findings suggest that the antimicrobial effect of included fillers will decrease over time. 5 If a long-term release is required, then recharge regimens are necessary. 18 Attempts to recharge the modified denture bases with fluoride succeeded and showed improved delayed fluoride release compared with initial values. 5

NaF has a high initial dissolution rate (1.581 µg/cm ^2^ after 7 days) that decreases drastically with time (0.05 µg/cm ^2^ after 14 days). 7 After 3 days only, fluoride release was two times lower than that at the first hour due to ease of dissolution of the compound and absence of chemical bond with PMMA. 7 Therefore, brief contact with water results in rapid disintegration and ion release. NaF- and 2-hydroxyethyl methacrylate (HEMA)-modified resin showed prolonged ion release for 28 days. In contrast, another study reported detectable levels of fluoride ions within the first week only. The discrepancies between the results might be due to different methodologies or the dispersion of the NaF fillers within the material without the protection of HEMA. 7 Zitz et al 48 evaluated the effect of incorporating CaF _2_ , NaF, and amine fluoride (AmF) in PMMA specimens on fluoride release after immersing in saliva. The highest ion release was linked to NaF-containing specimens while CaF _2_ -containing specimens showed a longer duration of action. These findings were explained based on the degree of solubility of the compounds. 48 G018–090 filler is a fluoride-releasing material that could be added to denture base resin to provide sustained fluoride ion release capable of preventing enamel demineralization. 8 Furthermore, controlling the pH of the solvent during this filler silanization process could maintain the fluoride concentration at the anticariogenic level for an extended duration. Thus, a novel fluoride-containing heat-polymerized acrylic resin would be an alternative approach to control caries in high caries-risk patients utilizing RPPs. 8

#### Mechanism of Action of Ions on Fungal cells

Antifungal therapies are required to inhibit fungal growth. Among the antifungal mechanisms reported is the creation of oxidative stress in *C. albicans* . Extraction liquids containing released ions-oxidative stress proved its effectiveness against *candida* adhesion, growth, biofilm formation, the transition from yeast-to-hyphae form, and proteinase production. 13 As a consequence, fluoride ions released from S-PRG fillers effectively create oxidative stress that is capable of inhibiting microbial growth and other pathogenic aspects of *C. albicans* , preventing candidiasis thereafter. 13 The mechanisms underlying the microbial antiadhesive property involve the interaction of fluoride ions with yeast membrane or the reduction of ergosterol content that destabilizes *Candida* membranes. 33 While on the other hand, sodium ions (from NaF) elevate the osmotic pressure, kill *C. albicans* cells, and increase the replication time. 23 For carriers containing silver-sulfadiazine (AgSD) compounds, the released silver ion denatures microbial deoxyribonucleic acid and ribonucleic acid to inhibit further cell duplication. Another mechanism of action of silver ions involves the inhibition of an enzyme important for cell wall synthesis (phosphomannose isomerase). 49

#### Drug Release

Drug release from modified PMMA produces long-term (weeks to months) and potent antifungal effects against *Candida* . The duration of drug release and percentage of *candida* reduction were directly correlated with loaded PNVP content. These results might be attributed to higher initial availability and greater sites for drug– *candida* interaction. 39 In the previous model, the modified PMMA was only a carrier for the antifungal drug, and the released drug disabled *Candida* cells preventing biofilm formation. 39 This unique interaction between carriers and antimicrobial drugs provides significant clinical implications when it comes to the management of DS.

PMMA acting as a carrier for different antimicrobial drugs showed different release rates depending on the loaded drug. A combination of two loaded drugs (5% chlorhexidine + 5% fluconazole) showed higher release rates in comparison to the single drug loading with a more potent antifungal effect. 45

The addition of MSNs to the PMMA matrix is valuable for the release of the loaded drugs (for example, amphotericin B). However, due to the breakdown of the MSN fillers during the biodegradation process, their long-term action or recharge is not guaranteed 14 but has been reported by some studies. 41 AgSD-MSNs added to PMMA release silver ions and sulfadiazine slowly into the environment to produce long-term antimicrobial activity. Note that 5 wt% loaded MSNs added to PMMA were able to improve hydrophilicity, deliver treatment drugs (i.e., amphotericin B), and show antimicrobial effects for an extended duration (14 days). 14

A novel HA-NP incorporated in PMMA and loaded with MZ has demonstrated continuous and steady release of MZ. In this study, the microbial reduction was dependent on drug concentration and diffusion capability, indicating that the leaching behavior of MZ into the surrounding environment was controlled by a concentration-dependent diffusion process. 22

#### Water Sorption and Solubility Role in Ion/Drug Release and Recharge

Physical properties of PMMA material such as water sorption and solubility play important roles in ions/drugs release and rechargeability. 7 11 50 PMMMA modified with 5 and 10% Kavitan or 10% Fritex complied with the International Organization for Standardization (ISO) 20795–1:2013 set values for water sorption (below 32 µm/mm ^3^ ) and solubility (1.6 µm/mm ^3^ ) of denture base materials. 7 However, the solubility of NaF-modified PMMA was 1.73 ± 0.15 µm/mm ^3^ , which might explain the high initial (7 days) release of fluoride ions. These findings confirm a higher dispersion rate of NaF within liquids and the absence of a chemical bond with PMMA. 7 Contrary to that are specimens modified with Kavitan and Fritex glass fillers, which showed slow initial release of ions that increased at day 14. 7 Similarly, PNVP-g-PMMA presented lower sorption and solubility capabilities than ISO standards, indicating a covalent bond between resin and grafted material, with absolute minimal material leach out. 39 On the other hand, miconazole and chlorhexidine digluconate loaded PNVP produced antifungal effects for months and weeks, respectively, indicating lower solubility of miconazole. 39 Similarly, PMMA loaded with fluconazole, chlorhexidine, or a combination of the two showed a sustained and steady drug release for up to 28 days. The sustained release of these drugs over time was linked to two mechanisms, rapid linear behavior following Flick's law, and discrete cluster formation around drug molecules. Additionally, drug release may have been potentiated by possible crack and porosity formation on the specimen's surface as a result of drug inclusion. 45

## Recharging Concept

The new resins containing ions or drug carriers slowly release antifungal agents over an extended period to prevent or treat fungal infections. For the high-risk patient (of DS), the filler-modified denture can be worn as an ordinary prosthesis in the absence of DS. However, if DS occurs, the same modified denture is loaded with antifungal agents during rest time to initiate antifungal therapy. When the infection resolves, the drug-loaded denture can be quenched to “wash out” residual bound drugs and suppress the therapeutic effects; returning the treatment denture to a normal denture. In the event of DS reoccurrence, a similar or different drug class can be used to recharge the denture and reinitiate the treatment. 39

### Recharge Mechanisms

#### Ions

Denture base resin and intracoronal restorations containing fluoride permitted a greater amount of ion release into the oral environment. What is important is not the sole release of fluoride but also the sustainability of its release through continuous recharge. 11 Therefore, the level of fluoride release and recharge depends on the bioactive glass filler concentration within the denture base resins/restorative material. Recent studies reported that S-PRG-modified PMMA fell into the category of materials with release/recharge capabilities. 5 11 Furthermore, the hydrophilicity of the PMMA is a key factor in water sorption and fluoride ion exchange (release and recharge). 11 Different protocols were suggested for fluoride recharge. 5 10 11 35 46 51 The fluoride release was reported when recharge involved the use of solution with 9,000 ppm, 5 however, this concentration of fluoride is not clinically applicable. 10 Another fluoride recharge mechanism was recommended by Xu and Burgess, 51 in which topical fluoride is painted onto the surface of the resin. Studies reported that high initial fluoride release is indicative of ease of recharge. 5 46 Also, recharging can be achieved by immersion of the specimens into a 5,000-ppm NaF gel for 5 minutes, 5 51 or having the patients follow a routine tooth brushing regimen. 35 To attain continuous fluoride release from a denture, a regular fluoride recharge protocol must be followed. 18

The attempt to charge a nonmodified PMMA denture base resin resulted in a short-acting effect of fluoride ions as it is washed away from the surface. On the other hand, fluoridated glass fillers in the modified PMMA acted as an ion reservoir and produced continuous release. This fact confirmed that not only modified denture bases could be recharged but also unmodified PMMA denture base materials have some degree of recharge capability. 11 When the rechargeability of pure and modified denture bases was compared, the latter absorbed more and released over a longer time, while the pure one absorbed and released fluoride easily and quickly, indicating that only the superficial ions were freed. 11

The degree of fluoride rechargeability of modified resin is influenced by the form and concentration of fluoride, protocol, duration, and frequency of fluoride exposure; regardless of the complexity or cost of the technique. 7 9 Kiatsirirote et al 18 reported that lower salivary pH increased the fluoride release as a result of the increased dissolution of the material. Kiatsirirote et al 18 reported optimal salivary fluoride release of S-PRG-modified resin upon daily recharge with 5,000 ppm fluoride gel. The S-PRG here worked as a reservoir of fluoride due to its structural nature and hydrogel content. 48 Fluoride-containing denture cleansing toothpaste could be a good source for recharge. 52 Ten percent Fritex-modified PMMA showed the ability to re-release fluoride ions into the oral cavity after an overnight recharge with a simple toothpaste solution. 7

#### Drugs

The incorporation of drug carriers within a denture base, allows the acrylic resin to absorb and slowly release the therapeutic drug. If infections reoccur or a prolonged duration of action is desired, a recharge process could take place. 39 The unique structure of MSNs (porous with large surface area) enables them to load and release drugs. 53 Because of that, the positive amino group of amphotericin B easily binds with the negatively charged nanomolecules to exert its antimicrobial effect. Amphotericin B had a prolonged duration of antimicrobial action (7 days) due to the slow release (1 ppm/day). Without drug loading, MSN-modified PMMA did not present any antimicrobial effects. 14

AgSD is a sustainable source of silver ions. It is a releasable effective antimicrobial drug that can be loaded into mesoporous MSN particles. 41 54 Its antimicrobial effect initiates after it decays into its forming element. 41 Because of its low solubility, the ions are released over an extended period providing a long-lasting antimicrobial effect. 54 Currently, there is an interest in incorporating AgSD-MSNs into denture base acrylic resin to produce long-lasting effects. 41 Prolonged antimicrobial effects (i.e., up to 28 days), are vital for some clinical situations. In this context, Jo et al 41 observed no further antimicrobial effects of specimens recharged with AgSD after 28 days of aging. Undeniably, the long-term properties of the modified materials with or without recharging using AgSD could be questionable. Grafting of PNVP onto the surface of denture resin was also investigated and suggested. 39 The grafted specimens can be charged with miconazole to slowly release it into the environment (> 1 month), then washed out to eliminate the remaining drug, and recharged again using a similar or different drug. However, investigators reported the reduction of miconazole release after 10 days; possibly due to protein contamination of the specimen surface. 40

## Effects of Ion Release on Modified Resin Properties

It has been proven that inorganic fluoride present in the particles within a denture base can dissolve and release into the environment. However, this process results in the formation of voids within the resin matrix that may affect its physical properties, but also permit deeper penetration of the recharge solution and enhance the potential for fluoride loading and release. 51 On the other hand, studies showed that fluoride induces corrosion of alloys, discolors β-titanium alloy wires, and damages titanium and titanium alloys at a concentration lower than half what is currently found in commercial dentifrices (< 453 ppm). 55 56

## Physical Properties of Rechargeable Denture Base (Mechanical Behavior and Surface Properties)

Although various additives increased the anticariogenic effect of the dental restoration, adverse effects of fluoridated filler may include decreased mechanical properties of the modified materials. 11 48 57 S-PRG decreased the mechanical properties (flexural strength) of the modified resin in a concentration-dependent fashion. However, the modified materials still complied with the ISO 1567 requirements except at higher concentrations (30% filler) or after prolonged aging. 5 11

Note that 20 wt% S-PRG-modified PMMA was not reported to have diminished mechanical properties over a study period of 1.5 years. 18 The literature has conflicting opinions about the level of filler inclusion. Kiatsirirote et al 18 recommended the inclusion of 20 wt% filler while Kamijo et al 5 and Al-Bakri et al 11 advocated the use of less than 20 and 10 wt% filler, respectively, to ensure maintaining the mechanical properties of the resin. When it comes to other fillers, Zitz et al 48 reported a decrease in bending strength of acrylic resin specimens after modifying with CaF _2_ , NaF, and AmF with the AmF having the greatest effect. Also, the addition of nonsilanized G018–090 fillers to PMMA still maintained the hydrophilic properties of the material resulting in high fluoride release. Upon fluoride release, excessive weak points are created leading to lower flexural strength. 8 If a silane coupling agent was used, it could prevent the hydrolysis and protect against filler leach out, which retains the material's mechanical properties for a longer period of time. 8

Conversely, some fillers showed promising results concerning the surface and mechanical properties of the modified material. Surface contact angle used as an indicator of the material's hydrophilicity/hydrophobicity showed a sharp decline in value after PNVP grafting, which confirms that the reaction of grafting took place on the surface of the resin. 39 Additionally, PMMA-g-PNVP resins met the minimum recommended flexural strength (65.0 MPa) and flexural modulus (2.0 GPa), suggesting that they could be used for clinical applications. 39 Similarly, the loading of MSNs into acrylic resin did not affect the mechanical properties significantly compared with the unmodified resin. 14 On the contrary, integrating Ag-MSNs into PMMA improved flexural properties in addition to surface hardness. 41 On the other hand, the free-surface energy of the MSN-modified specimens, showed a triple-fold increase when compared with nonmodified specimens. Most of the surface energy was in the form of polar force, suggesting a hydrophilic surface. Therefore, hydrophobic microbes, such as *C. albicans* and *Streptococcus oralis* , cannot readily adhere to the hydrophilic MSN-modified PMMA. 14

Similarly, filler addition increased the surface roughness. 3 However, contradicting results have been reported. It was stated that despite the increase in surface roughness of the modified specimens, they showed the least microbial adhesion of *C. albicans* and *S. mutans* , suggesting the continuous release of fluoride ions from the incorporated fillers. 3 7 On the contrary, other studies observed an increase in surface roughness after filler incorporation and the associated accelerated *candida* adhesion. 30 Thus, the resulting surface roughness at 20 wt% filler might have counteracted the antimicrobial effect of the released ions. 30 It is worth noting that the 10% filler was effective in reducing microbial adhesion, which highlights the negative effect of increasing the filler content on mechanical properties. 5 7 11 Five percent MSNs increased the surface roughness as a result of particle aggregation. 41

Hardness is a surface property that indicates the material's resistance to penetration and wear. MZ-loaded nanocarriers used to modify PMMA insignificantly decreased the surface hardness of the specimens. This observation might be linked to the disturbance of polymer-to-monomer ratio upon addition. The excessive unreacted monomer acts as a plasticizer that allows the deformation of the material under load. 22

Regardless of the change in surface and mechanical properties, the visual appearance and color of the S-PRG-modified resin were not easily distinguishable from the nonmodified one. 18

## Clinical Implementations

### Ions-Release Composite

Structural modification of removable denture material should either retain or improve the original mechanical properties of the material. 11 The amount of fluoride released should be sufficient to prevent tooth demineralization under the denture without causing toxicity. 5 Denture base resin can be recharged with fluoride by immersing the denture in a fluoride-containing cleanser when not in use. Wearing a fluoride-recharged denture will release fluoride into the oral environment to inhibit demineralization and enhance remineralization around abutment teeth and eventually prevent the development of carious lesions. 4 11 18 Fluoride can be incorporated into the dentures for geriatric patients or into bleaching trays for at-home bleaching, orthodontic retainers, or night guards, thus potentially improving the oral health of people of all ages. Finally, the animal model used to check the biocompatibility of fluoride-releasing acrylic resins concluded that the modified PMMA was biocompatible with a very mild tissue response. 58

### Drug-Loaded Composite

The excellent biocompatibility, hydrophilicity, and low toxicity of PNVP rendered it to be a widely used drug carrier for therapeutic purposes. 39 It was used to modify the surface of PMMA via plasma-initiated graft polymerizations to covalently bond it to the surface. It is unique that it does not dissolve out of the resin, resulting in superior physical properties of the modified denture material. 39 The new resin had a higher capability to absorb chlorhexidine digluconate or miconazole than the original acrylic resin and sustain their release for weeks or months producing very potent antifungal effects. MSN-modified PMMA that was loaded with amphotericin B showed lasting antimicrobial effects. Due to the convenient fabrication steps, the concept of drug loading of carriers and inclusion within the restorative material can be globalized on other chemically activated PMMA-based materials. An extra added benefit of MSN incorporation is rechargeability. This is granted due to the porous nature of MSNs as well as the low degradation rate. 14 41 Collectively, loaded MSNs can be effectively utilized in removable or provisional prosthodontic prostheses. 41 The novel HA-NP allowed the sustained release of MZ for over a month. An advantage of the HA-NP is the physical and chemical compatibility with the loaded drug as well as PMMA material. 22

The successful production of true antimicrobial PMMA that maintains the original mechanical properties is an ambitious idea but not yet a reality. Therefore, further investigations of all aspects (biological, mechanical, and esthetic) are required for different drugs/ions reloading before clinical applicability. Also, these investigations should focus on determining the efficacy of the modified materials in preventing caries and other oral infections in addition to reporting the appropriate filler concentration that would fulfill the clinical and financial needs of a patient. Furthermore, controlled clinical trials involving denture wearers with and without DS are essential to assess the antimicrobial effects of the fillers.

Among the limitations of this study is the inclusion of only one clinical trial that investigated the effects of fluoride release on demineralization and remineralization of abutment teeth while other included articles were limited to *in vitro* studies with their own limitations. Second, adequate comparison between the results was impaired due to the lack of standardization of methodology, analysis, and variation in the tested materials. However, considering the huge variation between the studies and the importance of the subject, this literature review was able to shed some light on the most current literature regarding the antimicrobial effects of different nano-modifiers of PMMA which might aid in designing future clinical trials.

## Conclusion

All ion/drug sources added to PMMA did not show cytotoxic effects. Incorporating ion and drug sources into PMMA removable prostheses and appliances is a promising antimicrobial method brought up by the release of incorporated ions and drugs for anticariogenic or microbial antiadhesive effects. In addition, the sustained release and recharge capacity of the incorporated fillers produced long-term antimicrobial dentures. These composites can inhibit tooth structure demineralization and enhance remineralization, as well as being a suitable method to prevent DS. Future studies are in demand to set the minimum inhibitory concentration of each antimicrobial agent and to further assess the biocompatibility before clinical use.
